# Supplementary material for: Genomic adaptation of giant viruses in polar oceans
Source: Nat Commun. 2023 Oct 12;14:6233. doi: 10.1038/s41467-023-41910-6 (PMC10570341; doi:10.1038/s41467-023-41910-6)
Supplement: Supplementary file 1 — Supplementary Information [file 41467_2023_41910_MOESM1_ESM.pdf]

Supplementary Information for

Genomic adaptation of giant viruses in polar oceans

Lingjie Meng, Tom O. Delmont, Morgan Gaïa, Eric Pelletier, Antonio Fernàndez-Guerra, Samuel Chaffron, Russell Y. Neches, Junyi Wu, Hiroto Kaneko, Hisashi Endo, Hiroyuki Ogata*

***Corresponding author**:

Hiroyuki Ogata (E-mail: ogata@kuicr.kyoto-u.ac.jp, Phone: +81-774-38-3270)

Supplementary Figures

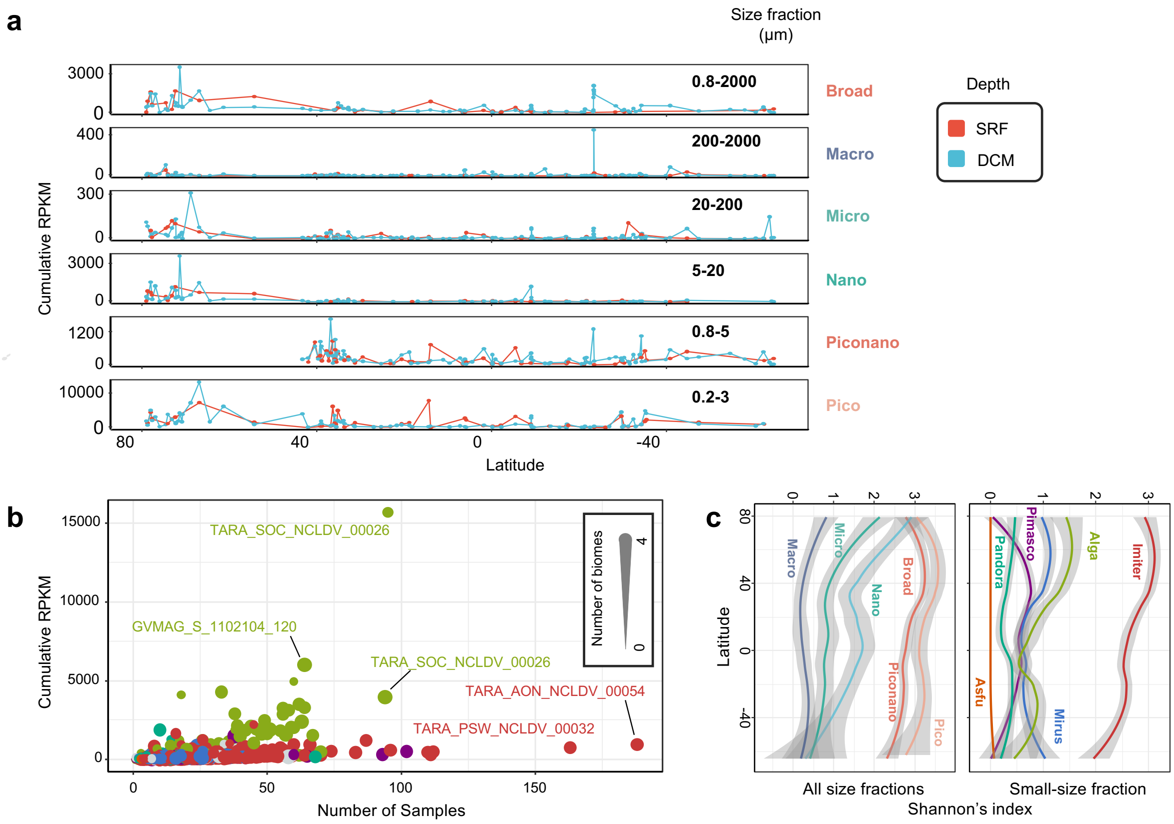

Supplementary Fig. 1 | Distribution of giant virus genomes. a, Cumulative coverage in each sample along the latitude. Individual size fractions are displayed separately, and line colours correspond to two depths (red for Surface, and blue for Deep Chlorophyll Maximum). **b,** Cumulative coverage of individual viral genomes versus the number of samples in which the

respective genomes were observed. The top five abundant/prevalent genomes are labelled. **c,** Locally estimated scatterplot smoothing plots of the latitudinal distributions of viral diversity (Shannon's index). The left panel presents the total diversity of all giant viruses along a latitudinal gradient in different size fractions. The Broad and Piconano size fractions were pooled because of their similar relative abundances and lack of Arctic samples in the Piconano size fraction. The right panel shows the diversity of communities of six main groups in the small-size fractions (Pico: 0.2–3 µm, Piconano: 0.8–5 µm, and Broad size: 0.8–2000 µm fractions).

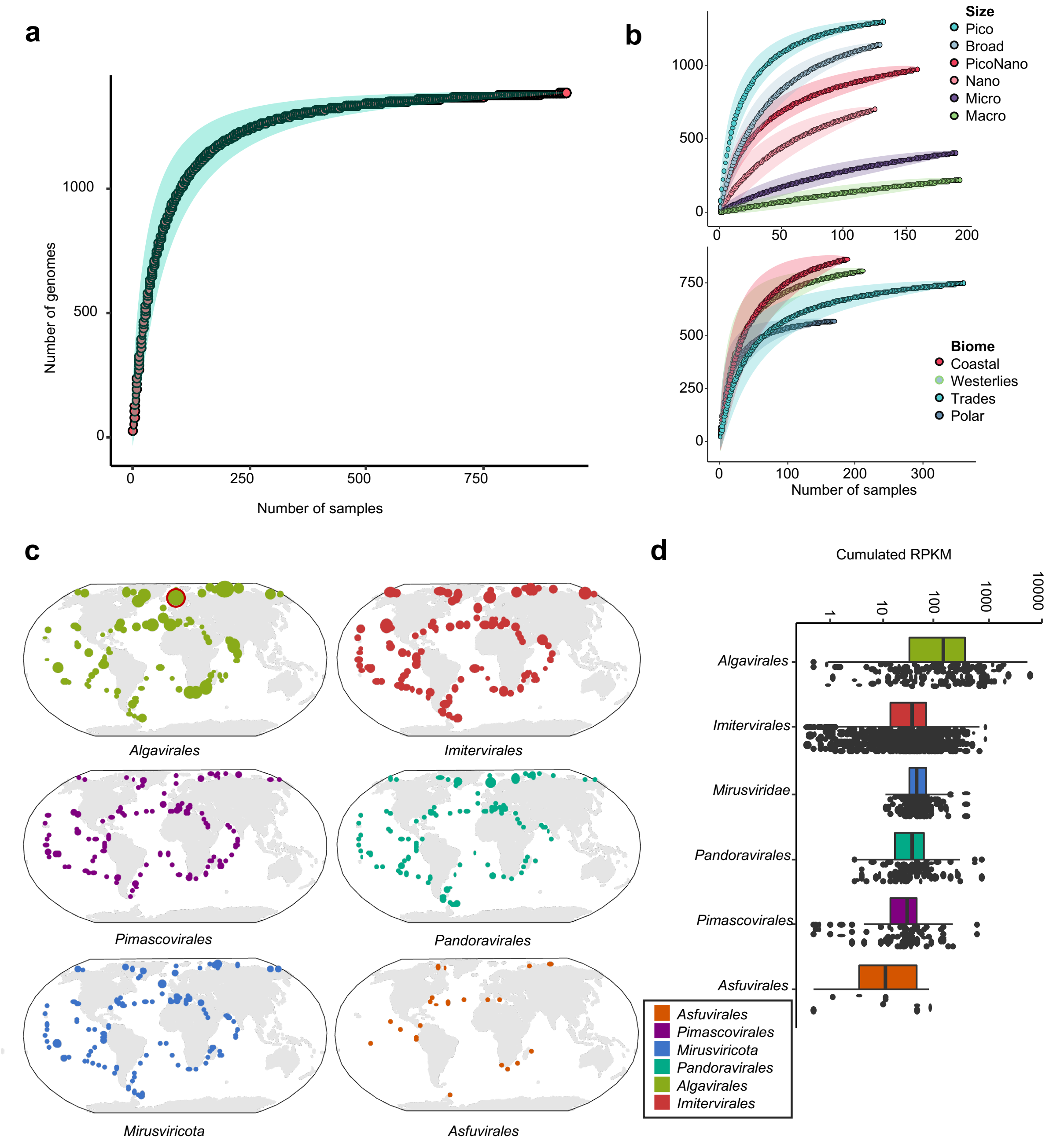

Supplementary Fig. 2 | Summary of the biogeography of viruses. a, Rarefaction curves for marine eukaryotic viral genomes in all samples and **b,** in subsamples separated by size fractions and biomes. For each curve, the average of 100 permutations is displayed with dots and the standard deviation is displayed with colour ranges. Rarefaction analyses reached a plateau

when all *Tara* Oceans samples were combined, but genomes in Micro (20–200 µm) and Macro (200–2000 µm) size fractions were still undersampled. **c,** Maps of the cumulative coverage of giant viruses in six main groups. Dot sizes are normalized by that of the most abundant dot, *Algavirales* in station 188 (marked with a red framed circle). **d,** Jitter box plots for the cumulative coverage of viral genomes classified into six main groups. There was a significant difference between groups ($P = 1.47\times10^{-21}$, Kruskal-Wallis test).

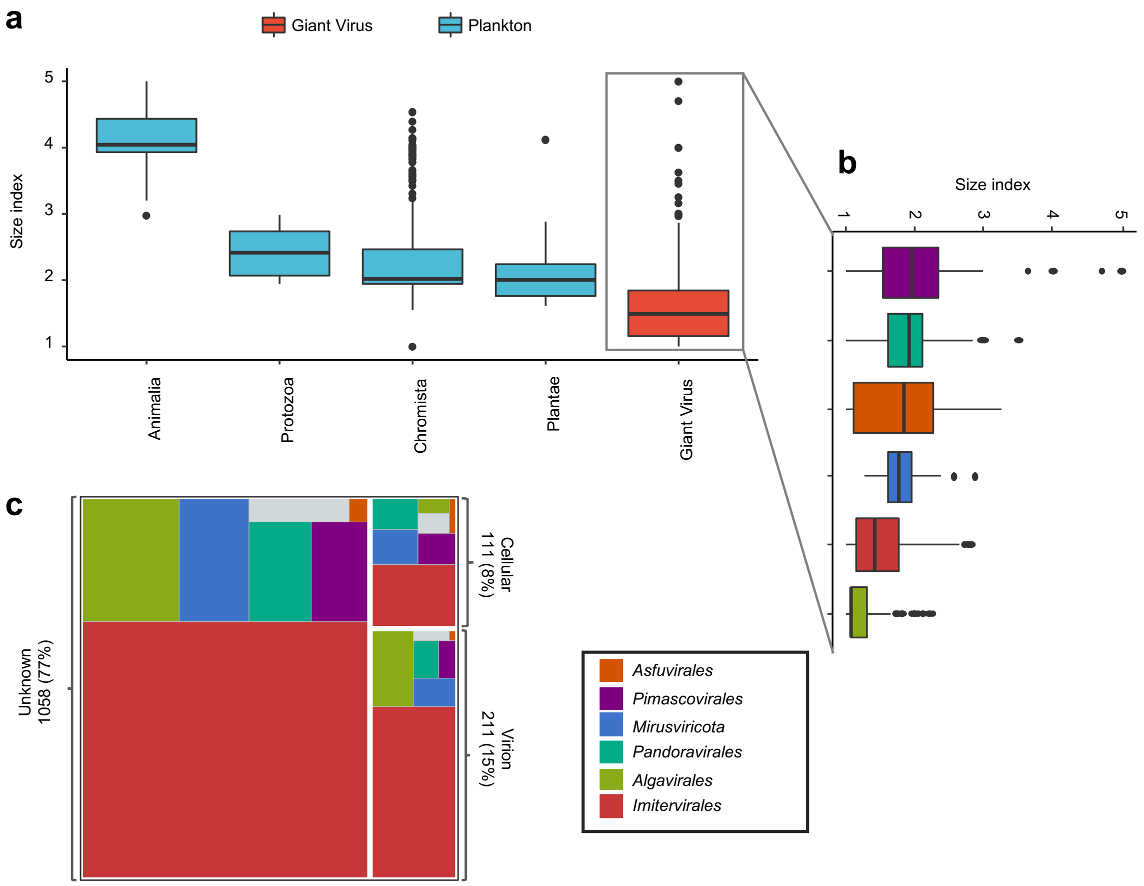

Supplementary Fig. 3 | Size distribution of viruses. a, Boxplots for the size indices of eukaryotic kingdoms and giant viruses. **b,** Boxplots for the size indices of six virus main groups. **c,** Treemap diagram showing the number of giant viruses assigned to "virion" or "cellular" size categories. Colours indicate the main groups.

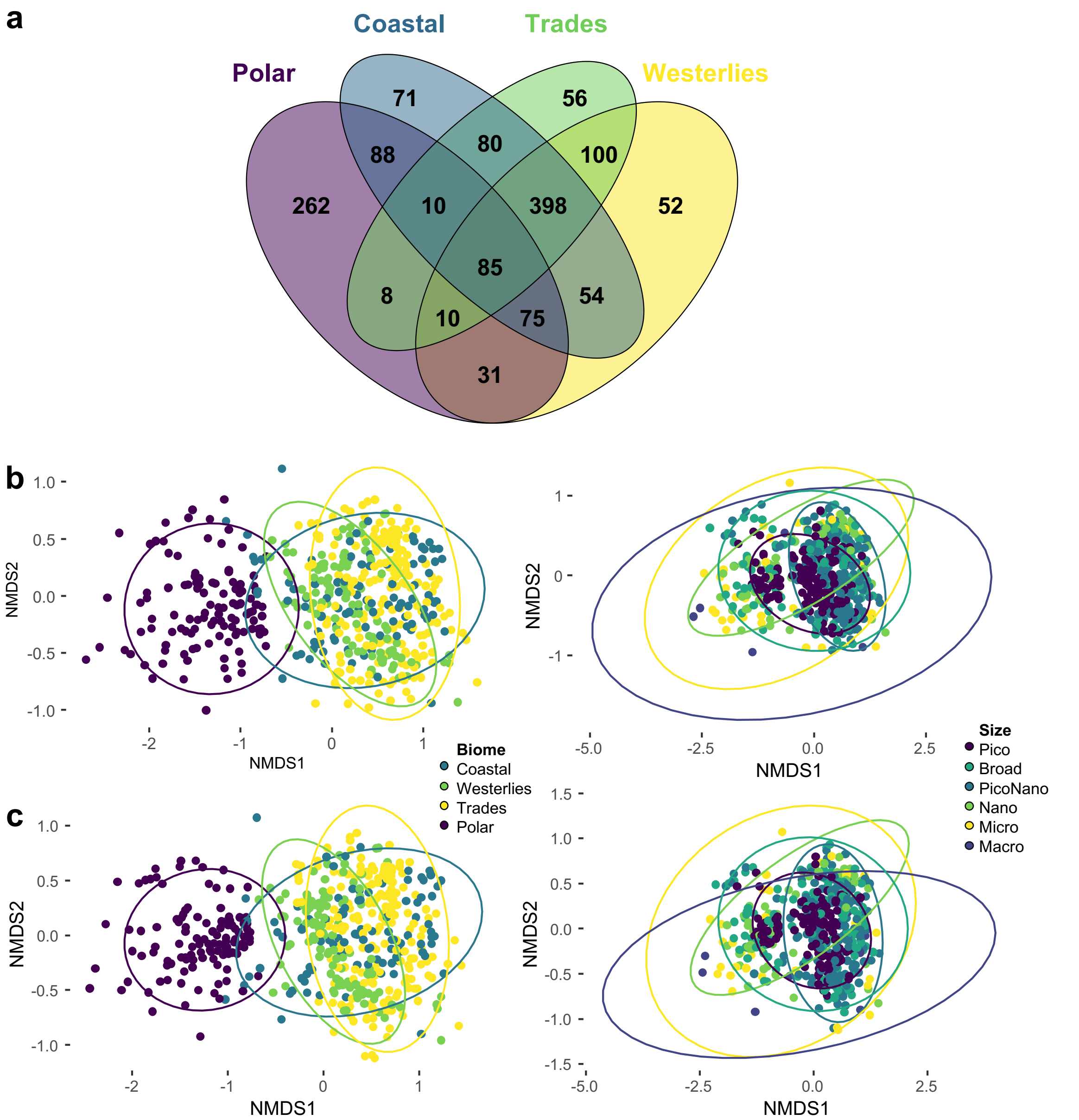

Supplementary Fig. 4 | Community structures of viruses. a, Venn diagram showing the numbers of shared or unique giant viruses across biomes. **b,c** NMDS ordination (Bray–Curtis dissimilarity) of the viruses communities for all samples using **(b)** relative abundance data (stress = 0.1617) and **(c)** presence/absence data (stress = 0.1541). Colours indicate biomes (left) and size fractions (right). Ellipses represent 95% confidence levels for each group. Statistical significance of the groupings was confirmed for both biome and size fraction groups (ANOSIM, $P < 0.01$)

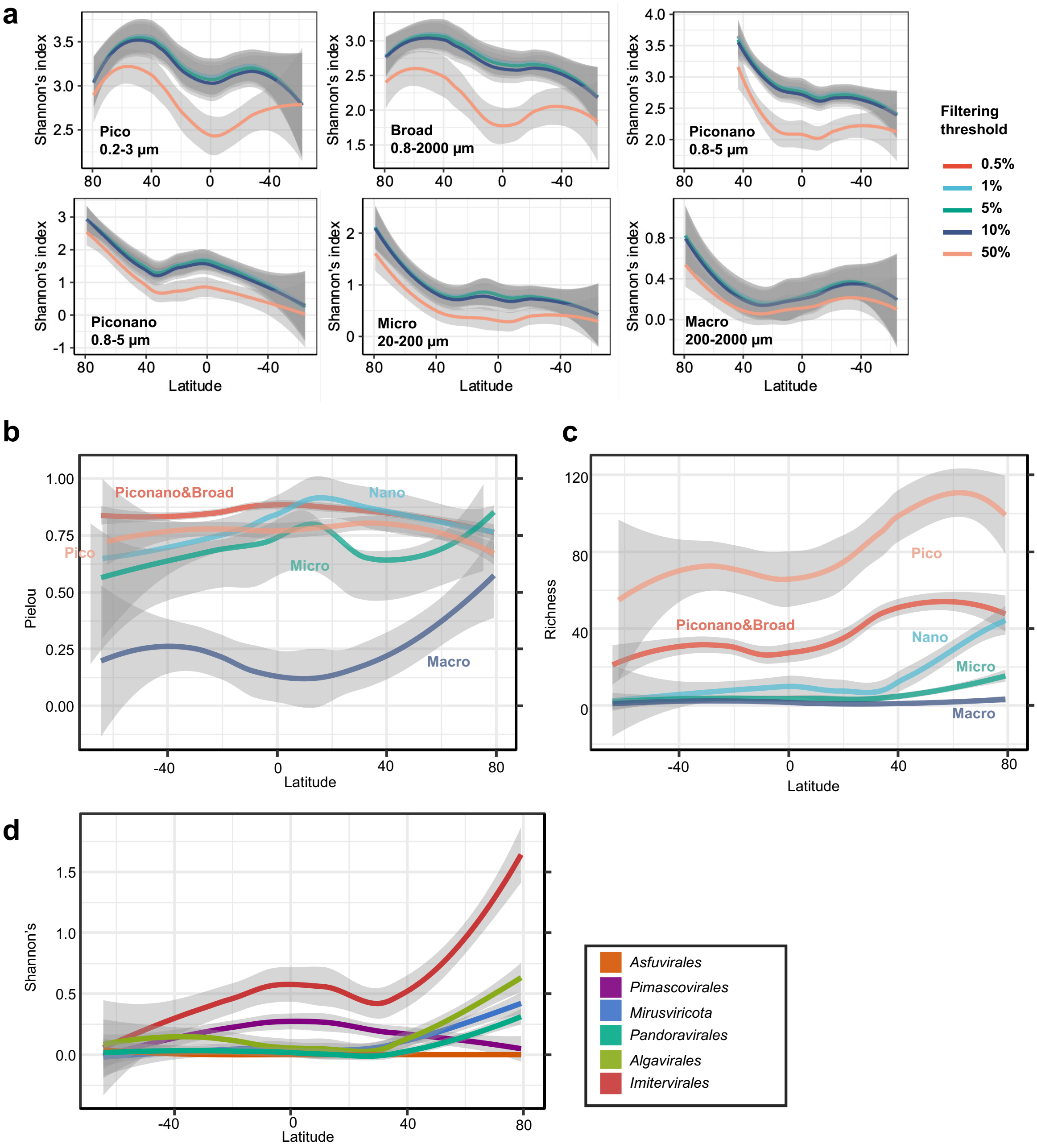

Supplementary Fig. 5 | Locally estimated scatterplot smoothing plots of the latitudinal distributions of viral communities. Latitudinal variation in **a,** Shannon's index of communities after trimming the lowest 0.5%, 1%, 5%, 10%, and 50% of the total number of non-zero RPKM values. **b,** Pielou's evenness and **c,** richness of marine viral communities for different size fractions. Colours indicate size fractions. **d,** Shannon's index of communities of six main groups in large-size fractions (Nano: 5-20 µm, Micro: 20-200 µm, and Macro: 200–2000 µm size fractions). Shaded areas represent 95% confidence intervals.

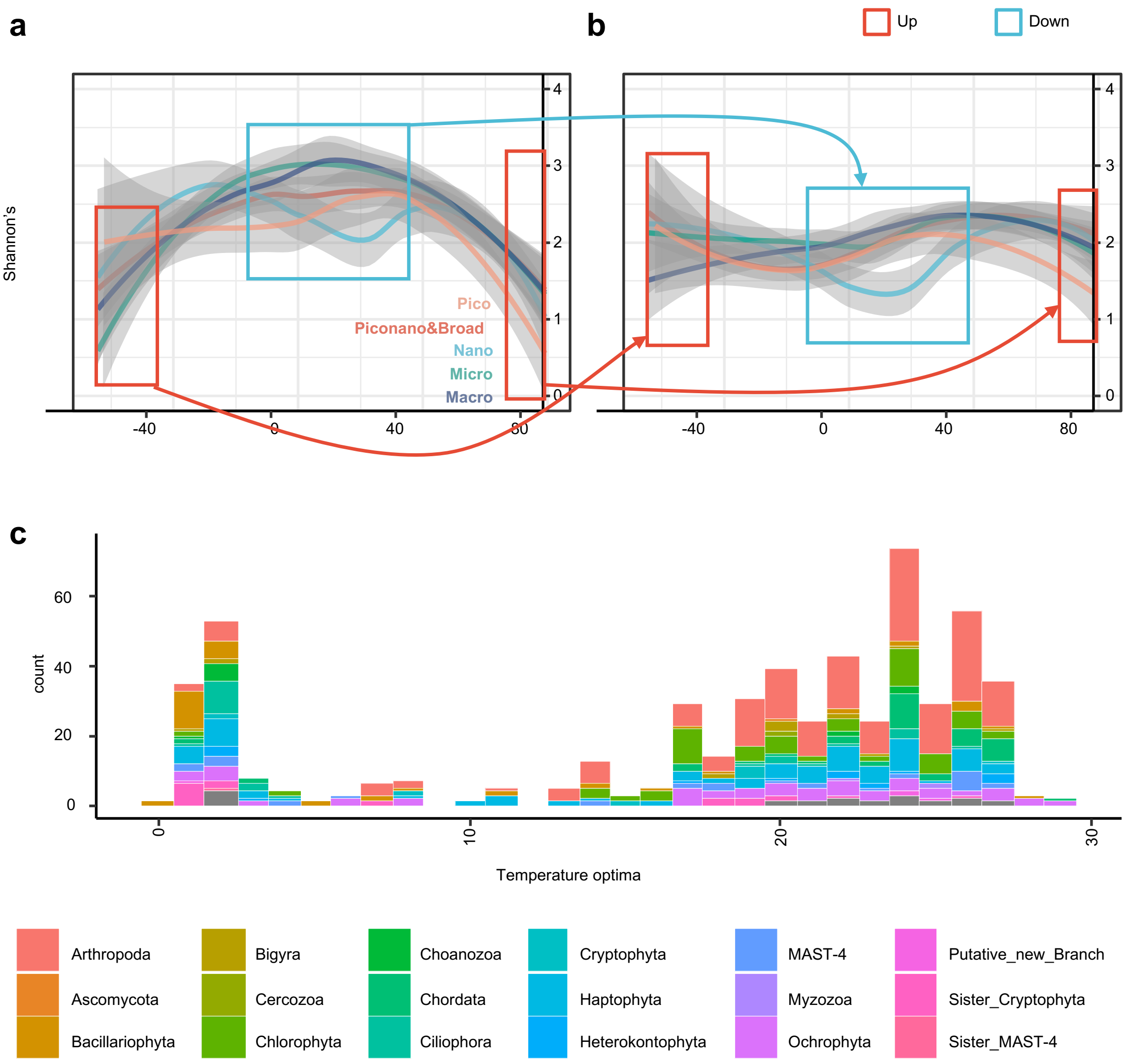

Supplementary Fig. 6 | Eukaryotic communities in the virus-host interactome. **a,b** Locally estimated scatterplot smoothing plots of the latitudinal distributions of eukaryotic community diversity (Shannon's index). **a,** Diversity of communities of eukaryotes that have no association with viruses in the network. **b,** Diversity of communities of eukaryotes that have associations with viruses in the network. Colours stand for size fractions. Polar regions and equatorial regions were marked with coloured frames. (C) Histogram of temperature optima of eukaryotic nodes. Colours represent the phylum of eukaryotic nodes as indicated at the bottom of the plots. Eukaryotic nodes with low temperature optima were enriched in diatoms (Bacillariophyta).

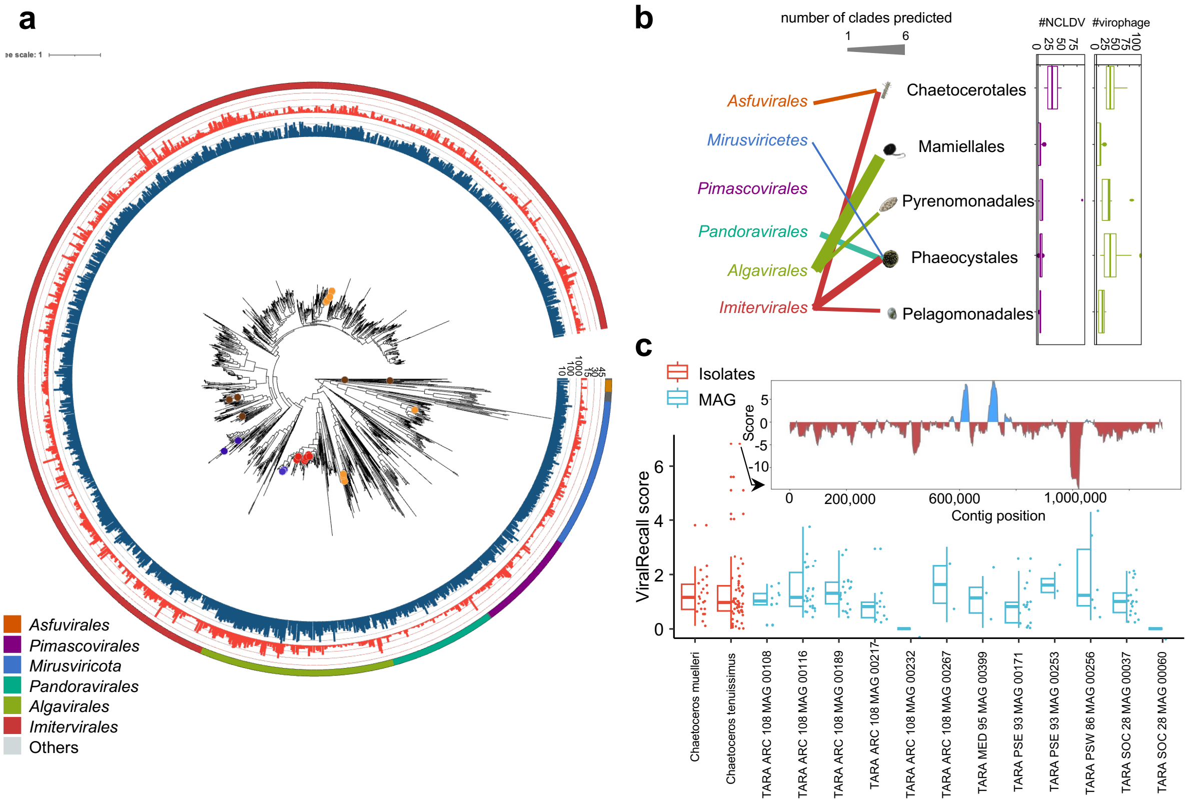

Supplementary Fig. 7 | Host prediction of viruses. a, Phylogenetic tree based on four hallmark genes with putative host groups (coloured circles) predicted by TIM, which are summarized in (**b**). The outermost layer shows the taxonomy of six main groups. The middle and inner layers show the number of stations in which the viruses were observed and cumulative coverage, respectively. **b,** Summary of host prediction results. Left panel: line colours represent the six main groups; line widths are proportional to the number of clades predicted to the associated hosts. Right panel: boxplots show the number of viral insertions detected in predicted host genomes. **c,** ViralRecall scores of 12 Chaetocerotales MAGs and 2 isolates. An example of *Chaetoceros tenuissimus* contig was given. Viral scores were evaluated with a rolling window of 15 ORFs on a contig. Positive and negative scores represent viral and cellular regions, respectively.

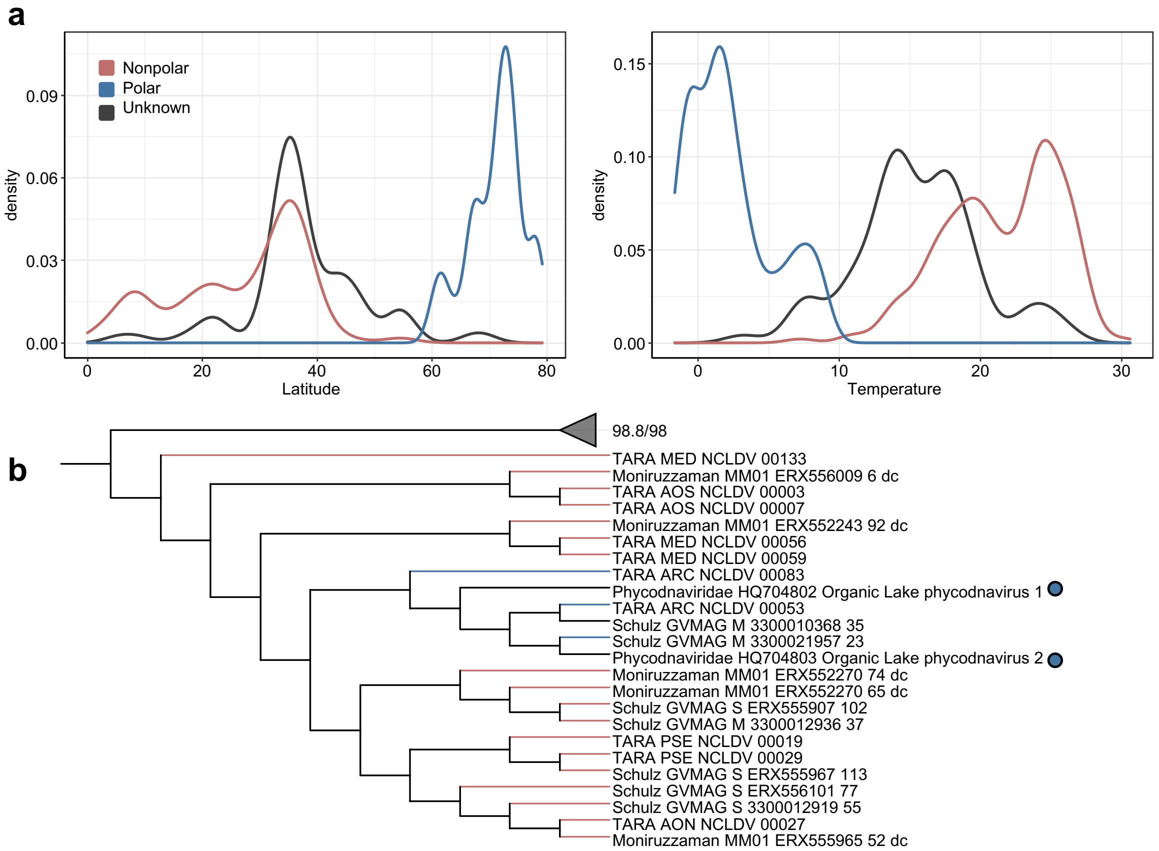

Supplementary Fig. 8 | Niche assignments of viral genomes. a, Density plots of the latitude and temperature optima. Three biome assignment groups (Polar, Nonpolar, and Unknown) are displayed separately. **b,** The Polar clade containing the reference Organic Lake phycodnaviruses (blue circles). Ancestral states of Nonpolar and Polar were estimated using the phylogenetic tree based on a one-parameter equal rates model. Blue stands for Polar and red stands for Nonpolar viruses.

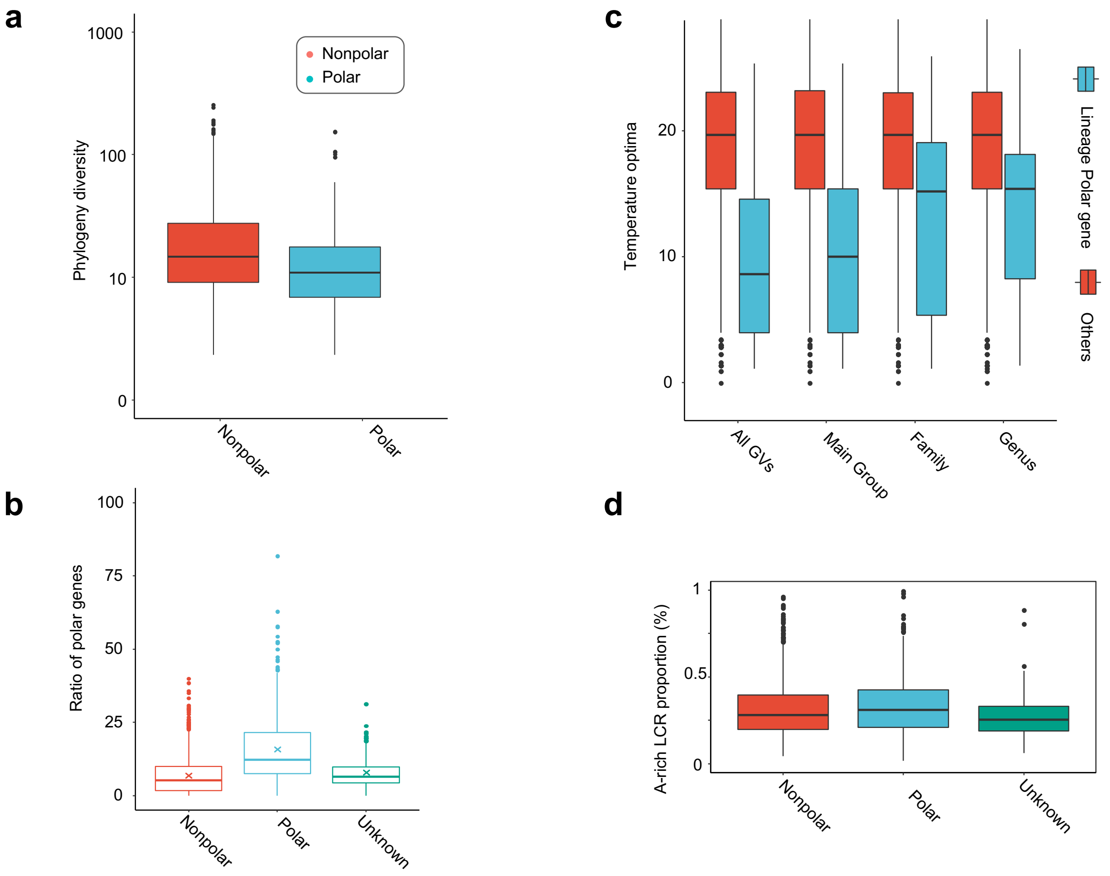

Supplementary Fig. 9 | Polar-specific KOs in polar viral genomes. a, Phylogenetic diversity of Polar-specific and Nonpolar-specific KOs. **b,** Ratio of Polar-specific KOs in Polar, Nonpolar and biome-unknown genomes. **c,** Boxplots of the temperature optima of Polar KOs enriched in Polar viral genomes at least one lineage (blue) and other KOs (KOs that were not enriched in Polar viral genomes at any lineage at four taxonomy levels, i.e., root, main group, family, and genus) (red). Enrichment analyses were performed at the main group, family, and genus levels. **d,** Alanine(A)-rich low-complexity regions (LCR) proportion in Polar, Nonpolar and biome-unknown genomes.

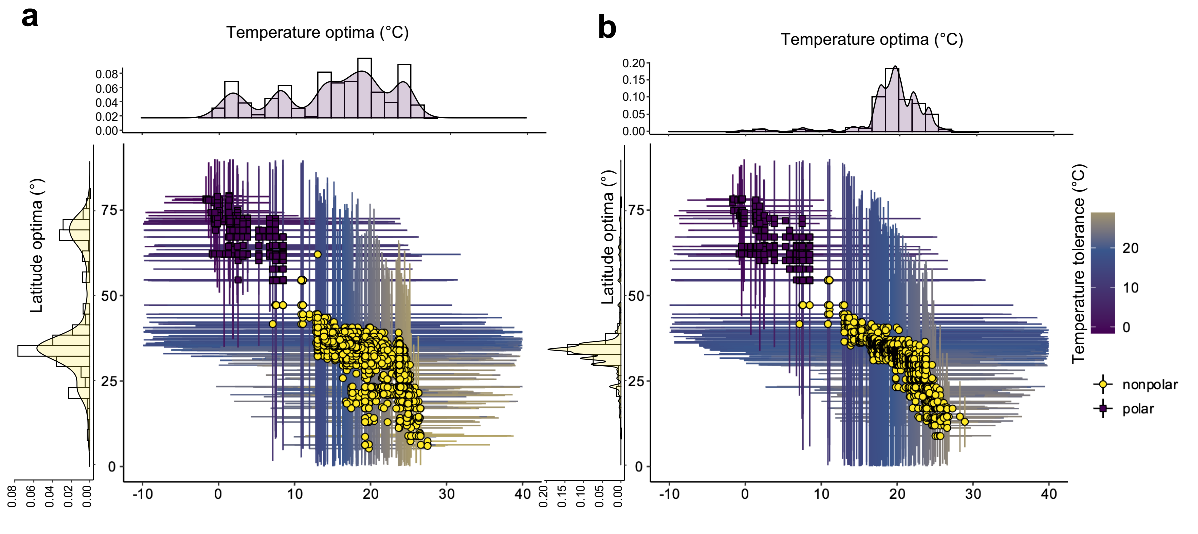

Supplementary Fig. 10 | Distributions of robust ecological optima for the orthologous groups of viruses and KOs in eukaryotic genomes. a, Scatterplot of the temperature and latitude optimum of AGNOSTOS gene cluster communities (GCCs) for viruses. Bars indicate the tolerance ranges of temperature (horizontal) and latitude (vertical). Histograms show the distributions of the temperature and latitude optima of GCCs. **b,** Distribution of temperature and latitude optima of KOs of eukaryotic genomes.

Supplementary Discussion

Biogeography of giant virus genomes

We used giant virus genomes from the Global Ocean Eukaryotic Viral (GOEV) database[1]. The initial version of the GOEV database included 698 genomes reconstructed from 798 *Tara Oceans* metagenomes, 1,187 metagenome-assembled genomes (MAGs) from two previous metagenomic surveys[2,3], and 235 reference NCLDV genomes. We eliminated redundancy by implementing a cut-off with Average Nucleotide Identity (ANI) of 98%, ultimately resulting in a refined database containing 1,817 genomes. The abundance profiles of 1,817 genomes across *Tara* Oceans samples from different size fractions (Fig. 1a; Supplementary Fig. 1a; Supplementary Data 1) revealed 1,380 viral genomes that showed signals in at least one sample out of 928 samples (see Methods). Rarefaction analyses showed reached a plateau when all *Tara* Oceans samples are combined, but genomes in micro- (20-200 µm) and macro-size (200-2000 µm) fractions were still under-sampled (Supplementary Fig. 2a,b). Detected viruses were

taxonomically classified into six main groups based on our phylogenomic analysis, with genome sizes ranging from 50 Kb to 1.6 Mb: *Algavirales* (n = 155), *Asfuvirales* (n = 9), *Imitervirales* (n = 913), *Pandoravirales* (n = 81), *Pimascovirales* (n = 75), and *Mirusviricota* (n = 111). Of these, all but the *Mirusviricota* belong to the *Nucleocytoviricota* phylum of the realm *Varidnaviria*. *Mirusviricota* belongs to the realm *Duplodnaviria* (Supplementary Data 2). These viruses, recently identified, possess indeed the morphogenetic module of this realm, even though a much larger fraction of informational genes relates them to *Nucleocytoviricota*[4].

In agreement with previous results based on marker genes[5,6], imiterviruses formed the largest group, with some members distributed widely (>100 samples) but at relatively low abundance (Supplementary Fig. 1b; Supplementary Fig. 2c,d). The cumulative RPKM (i.e., sum of the RPKM in all samples for a given genome) in the second largest group, algaviruses, was significantly higher than for other groups (Kruskal-Wallis test, $P = 1.47\times10^{-21}$) but these viruses were observed in fewer samples (≤95 samples) than *Imitervirales* (Supplementary Fig. 2c; Supplementary Fig. 2d).

Distribution of giant viruses across size fractions

Most previous metagenomic surveys on giant viruses analysed data from a pico-size fraction (0.2–3.0 µm)[7,8]. Our dataset allowed us to assess abundance of viruses in larger size fractions up to the meso-fraction (200–2000 µm). Giant viruses were detected in both small and large size fractions at many stations (Supplementary Fig. 1a). Accordingly, size index values (i.e., a measure of sampling size fraction preference for each genome) were distributed widely for giant viruses compared with those of individual eukaryotic taxa (Supplementary Fig. 3a,b). Viral signals in large size fractions (e.g., >0.8 µm) may originate from viral genomes inside their host cells, whereas those in small size fractions (e.g., 0.2–3.0 µm) may originate from viral genomes from either free virions or within host cells.

We assigned infection stage categories to individual viral genomes [either “virion” (0.2–3.0 μm) or “cellular” (>0.8 μm)] based on the reads per kilobase of genome per million reads mapped (RPKM) distribution across size fractions (Supplementary Fig. 3c; Supplementary Data 2). This categorization demonstrated that 15% of viruses (n = 211) were over-represented in the “virion” category, while 8% (n = 111) were over-represented in the “cellular” category. The proportions of *Imitervirales* and *Algavirales* were relatively low, and those of *Pimascovirales*, *Pandoravirales*, *Asfuvirales* and *Mirusviricota* were relatively high for the “cellular” category compared with those in the “virion” category, implying different host size ranges for different groups of viruses.

Impact of environmental variables on latitudinal distribution pattern

We estimated robust ecological optimum (temperature, salinity, latitude, ChlorophyllA, Si, NO2, PO4) for individual viral genomes (Supplementary Data 2). We performed a Spearman analysis to understand the correlation between latitude and the different environmental optima. Temperature was found most correlated with the latitude (Spearman rho = -0.886), which was followed by salinity (rho=-0.432) and ChlorophyllA (rho=0.579). No other variable had an absolute rho higher than 0.1. Moreover, neither salinity nor ChlorophyllA appear to distinctly drive the virus-eukaryote network like Temperature does (Figure 1b). Therefore, we considered temperature is the most appropriate variable to explain the latitudinal (i.e., Polar vs Nonpolar) distribution of viruses in this study.

Supplementary References

1. Gaïa, M. *et al.* Mirusviruses link herpesviruses to giant viruses. *Nature* 1–7 (2023).
2. Schulz, F. *et al.* Giant virus diversity and host interactions through global metagenomics. *Nature* **578**, 432–436 (2020).
3. Moniruzzaman, M., Martinez-Gutierrez, C. A., Weinheimer, A. R. & Aylward, F. O. Dynamic genome evolution and complex virocell metabolism of globally-distributed giant viruses. *Nat Commun* **11**, 1–11 (2020).
4. Gaïa, M., Meng, L., Pelletier, E., Forterre, P. & Vanni, C. Plankton-infecting relatives of herpesviruses clarify the evolutionary trajectory of giant viruses. (2022).
5. Li, Y. *et al.* Degenerate PCR primers to reveal the diversity of giant viruses in coastal waters. *Viruses* **10**, 496 (2018).
6. Endo, H. *et al.* Biogeography of marine giant viruses reveals their interplay with eukaryotes and ecological functions. *Nat Ecol Evol* **4**, 1639–1649 (2020).
7. Hingamp, P. *et al.* Exploring nucleo-cytoplasmic large DNA viruses in Tara Oceans microbial metagenomes. *ISME Journal* **7**, 1678–1695 (2013).
8. Endo, H. *et al.* Biogeography of marine giant viruses reveals their interplay with eukaryotes and ecological functions. *Nat Ecol Evol* **4**, 1639–1649 (2020).
