# Supplementary material for: Genomic adaptation of giant viruses in polar oceans
Source: Nat Commun. 2023 Oct 12;14:6233. doi: 10.1038/s41467-023-41910-6 (PMC10570341; doi:10.1038/s41467-023-41910-6)
Supplement: Supplementary file 3 — Description of Additional Supplementary Files [file 41467_2023_41910_MOESM3_ESM.pdf]

Description of Additional Supplementary Files

Title: Supplementary Data 1
Description: Information of 928 metagenomes from the GOEV database. It provides sample identifiers accompanied by the respective sampling station and depth. These samples have various size fractions, which are detailed as follows: Size_P: 0.22–1.6 µm or 0.22–3.0 µm, Size_All: >0.22 µm, Size_01: 0.8–5 µm, Size_02: 5–20 µm, Size_03: 20–200 µm, Size_04: 200–2,000 µm.

Title: Supplementary Data 2
Description: Information of 1,380 genomes and their contigs in the GOEV database. The data are divided into four individual sheets. The first sheet contains the accession numbers for all genomes and MAGs listed in the GOEV database. The second sheet provides genomic and ecological information on the 1,380 genomes. The third sheet provides the gene content of each contig, detailing the number of gene origins. The fourth sheet highlights the gene origin of contigs that encode three specific KOs (K18589, K02575, K00720) as mentioned in the manuscript.

Title: Supplementary Data 3
Description: Marine eukaryote and virus cooccurrence network. For each association, eukaryotic names, eukaryotic orders, viral names, and viral main groups are provided. The network is pooled with associations from five size fractions, retaining only the best positive or negative associations (i.e., the edges with the highest absolute weights).

Title: Supplementary Data 4
Description: Information of functions (KOs). The first sheet contains ecological information of each KO. The second sheet lists the top 20 enriched KOs for different taxonomic levels. The fraction of components (i.e., enzymes) defined as polar-specific KOs was compared with the fraction of all other pathways, listed in the third sheet. This fraction was tested by Fisher's exact test and adjusted by the Benjamini-Hochberg (BH) correction. The result is given in the third sheet.
