# Supplementary material for: Genomic adaptation of giant viruses in polar oceans
Source: Nat Commun. 2023 Oct 12;14:6233. doi: 10.1038/s41467-023-41910-6 (PMC10570341; doi:10.1038/s41467-023-41910-6)
Supplement: Supplementary file 8 — Reporting Summary [file 41467_2023_41910_MOESM8_ESM.pdf]

nature portfolio

Corresponding author(s): Hiroyuki Ogata

Last updated by author(s): Aug 30, 2023

Reporting Summary

Nature Portfolio wishes to improve the reproducibility of the work that we publish. This form provides structure for consistency and transparency in reporting. For further information on Nature Portfolio policies, see our Editorial Policies and the Editorial Policy Checklist.

Statistics

For all statistical analyses, confirm that the following items are present in the figure legend, table legend, main text, or Methods section.

n/a	Confirmed	
☐	☒	The exact sample size (*n*) for each experimental group/condition, given as a discrete number and unit of measurement
☒	☐	A statement on whether measurements were taken from distinct samples or whether the same sample was measured repeatedly
☐	☒	The statistical test(s) used AND whether they are one- or two-sided<br>*Only common tests should be described solely by name; describe more complex techniques in the Methods section.*
☐	☒	A description of all covariates tested
☐	☒	A description of any assumptions or corrections, such as tests of normality and adjustment for multiple comparisons
☐	☒	A full description of the statistical parameters including central tendency (e.g. means) or other basic estimates (e.g. regression coefficient) AND variation (e.g. standard deviation) or associated estimates of uncertainty (e.g. confidence intervals)
☐	☒	For null hypothesis testing, the test statistic (e.g. *F*, *t*, *r*) with confidence intervals, effect sizes, degrees of freedom and *P* value noted<br>*Give P values as exact values whenever suitable.*
☒	☐	For Bayesian analysis, information on the choice of priors and Markov chain Monte Carlo settings
☒	☐	For hierarchical and complex designs, identification of the appropriate level for tests and full reporting of outcomes
☐	☒	Estimates of effect sizes (e.g. Cohen's *d*, Pearson's *r*), indicating how they were calculated

Our web collection on statistics for biologists contains articles on many of the points above.

Software and code

Policy information about availability of computer code

Data collection	no software was used to collect data
Data analysis	BWA v0.7.15, IQ-TREE v.1.6.2, ETE3 toolkit v.3.1.1, iTOL v.6, R v.4.0.1, Rstudio v.1.3.959, Prodigal v.2.6.3, anvi'o v6.1, eggNOG-mapper v.2.1.5, InterProScan v.5.44-79.0, usearch v.11.0.667, Cytoscape v.3.7.1, Diamond v.2.0.6, TIM, VirSorter2 v.2.2.3, ViralRecall v.2.1, SciPy v.1.7.1<br>We used code and a Github repository to calculate robust ecological optima, which can be accessed at https://github.com/LingjieEcoEvo/PolarAdaptaiton/tree/main/optimum.

For manuscripts utilizing custom algorithms or software that are central to the research but not yet described in published literature, software must be made available to editors and reviewers. We strongly encourage code deposition in a community repository (e.g. GitHub). See the Nature Portfolio guidelines for submitting code & software for further information.

Data

Policy information about availability of data

All manuscripts must include a data availability statement. This statement should provide the following information, where applicable:

- Accession codes, unique identifiers, or web links for publicly available datasets
- A description of any restrictions on data availability
- For clinical datasets or third party data, please ensure that the statement adheres to our policy

The Tara Oceans metagenome data is accessible at ENA under the accession PRJEB402. Databases in this study include: The FASTA files for the 1,380 giant virus

genomes from the Global Ocean Eukaryotic Viral (GOEV) database (https://doi.org/10.6084/m9.figshare.20284713); Giant Virus Orthologous Groups (GVOGs) database (https://zenodo.org/record/4762520/files/hmm.tar.gz); Virus-Host Database (https://www.genome.jp/virushostdb); Tara Oceans Eukaryotic Genomes Database (https://www.genoscope.cns.fr/tara); NCBI database (https://www.ncbi.nlm.nih.gov/genome) . The data utilized in this study can be accessed from GenomeNet at https://www.genome.jp/ftp/db/community/tara/PolarAdaptaiton/data/. Source data are provided with this paper.

Research involving human participants, their data, or biological material

Policy information about studies with human participants or human data. See also policy information about sex, gender (identity/presentation), and sexual orientation and race, ethnicity and racism.

Reporting on sex and gender	Not applicable
Reporting on race, ethnicity, or other socially relevant groupings	Not applicable
Population characteristics	Not applicable
Recruitment	Not applicable
Ethics oversight	Not applicable

Note that full information on the approval of the study protocol must also be provided in the manuscript.

Field-specific reporting

Please select the one below that is the best fit for your research. If you are not sure, read the appropriate sections before making your selection.

☐ Life sciences ☐ Behavioural & social sciences ☒ Ecological, evolutionary & environmental sciences

For a reference copy of the document with all sections, see nature.com/documents/nr-reporting-summary-flat.pdf

Ecological, evolutionary & environmental sciences study design

All studies must disclose on these points even when the disclosure is negative.

Study description	The study is based on metagenomic data generated by the Tara Oceans consortium over the years. We investigated genomes of eukaryotic large DNA viruses to characterize the viral genome-level adaptation to polar environments.
Research sample	Sunlit oceans (Tara Oceans). Thank you for raising this point. The choice of samples in our study was for ensuring the diversity of viral communities at a global scale. 924 samples from Tara Oceans encompass different geographical locations/biomes, which could help people understand the biogeography of giant viruses. The samples we selected aim to represent the viral communities of giant viruses, as well as their potential hosts.
Sampling strategy	The study did not involve any sampling. We used data generated by the Tara Oceans consortium. The selection of datasets for this study was informed by findings from earlier research. Past studies revealed that the abundance of giant viruses is mainly detected within the pico-size fraction of Tara Oceans metagenomic data. Concurrently, a significant proportion of their host cells span a size fraction ranging from 0.8-2000 µm. With this insight, to capture a comprehensive spectrum of giant viruses, whether "free-living" or in an infecting state, we opted to incorporate datasets (924 metagenomes) from both the Tara Oceans prokaryotic and plankton size fractions.
Data collection	We utilized all Tara Oceans metagenomes for the reasons previously mentioned. These metagenomes were generated for earlier publications and are archived at the ENA under accession PRJEB402. Tom O. Delmont accessed and downloaded this public data to create the Global Ocean Eukaryotic Viral (GOEV) database. The Tara Oceans metagenomes and GOEV database are currently recorded in both the Genoscope and Kyoto University supercomputer systems. Additionally, the data, which includes both the raw metagenomes and the GOEV database, is publicly accessible at https://doi.org/10.6084/m9.figshare.20284713.
Timing and spatial scale	The Tara Oceans sampling began on September 1st, 2009, and concluded on March 18th, 2012. A year later, the Tara Arctic sampling project commenced on June 3rd, 2013, and wrapped up on October 27th, 2013. The sampling spanned four primary marine biomes: Westerlies, Coastal, Trades, and Polar, covering a latitude range of 143°.
Data exclusions	No data was excluded.
Reproducibility	All data is available, and the tool is available for all to reproduce our findings.
Randomization	The sampling points of Tara Oceans were not randomly chosen. However, the Tara Oceans consortium has taken several measures to control the covariates. Firstly, Tara Oceans utilized standardized sampling protocols and equipment, ensuring consistency in all samples across different biomes and time points. Secondly, Tara Oceans sampling locations were selected to maximally cover a wide range of ecosystems through the optical data from satellites. Thirdly, Tara Oceans' sampling spanned different seasons, months, dates, and times for each biome, which ensures that the observed biogeographical patterns aren't a mere reflection of the sampling season or date. Alongside biological samples, Tara Oceans also gathered detailed physical and chemical data. These measurements help control for various environmental gradients when analyzing biological data.

Blinding | We worked on all the metagenomes of Tara Oceans, so their is no blinding.

Did the study involve field work? ☐ Yes ☒ No

Reporting for specific materials, systems and methods

We require information from authors about some types of materials, experimental systems and methods used in many studies. Here, indicate whether each material, system or method listed is relevant to your study. If you are not sure if a list item applies to your research, read the appropriate section before selecting a response.

Materials & experimental systems

n/a	Involved in the study	
☒	☐	Antibodies
☒	☐	Eukaryotic cell lines
☒	☐	Palaeontology and archaeology
☒	☐	Animals and other organisms
☒	☐	Clinical data
☒	☐	Dual use research of concern
☒	☐	Plants

Methods

n/a	Involved in the study	
☒	☐	ChIP-seq
☒	☐	Flow cytometry
☒	☐	MRI-based neuroimaging
